# Sertoli cell and spermatogonial development in pigs

**DOI:** 10.1186/s40104-022-00687-2

**Published:** 2022-04-11

**Authors:** Yi Zheng, Qiang Gao, Tianjiao Li, Ruifang Liu, Zechao Cheng, Ming Guo, Jinhong Xiao, De Wu, Wenxian Zeng

**Affiliations:** 1 Key Laboratory for Animal Genetics, Breeding and Reproduction of Shaanxi Province, College of Animal Science and Technology, Northwest A&F University, Yangling, 712100 Shaanxi China; 2 Zhumei Porcine Breeding Corporation, Zhengzhou, Henan China; 3 Key Laboratory for Animal Disease Resistance Nutrition of the Ministry of Education of China, Institute of Animal Nutrition, Sichuan Agricultural University, Chengdu, 611100 Sichuan China

**Keywords:** Pig, Sertoli cell, Spermatogenesis, Spermatogonia, Testis

## Abstract

**Background:**

Spermatogenesis is an intricate developmental process during which undifferentiated spermatogonia, containing spermatogonial stem cells (SSCs), undergo self-renewal and differentiation to generate eventually mature spermatozoa. Spermatogenesis occurs in seminiferous tubules within the testis, and the seminiferous tubules harbor Sertoli and germ cells. Sertoli cells are an essential somatic cell type within the microenvironment that support and steer male germ cell development, whereas spermatogonia are the primitive male germ cells at the onset of spermatogenesis. While the developmental progression of Sertoli cells and spermatogonia has been well established in mice, much less is known in other mammalian species including pigs.

**Results:**

To acquire knowledge of Sertoli cell and spermatogonial development in pigs, here we collected as many as nine ages of Duroc porcine testes from the neonate to sexual maturity, i.e., testes from 7-, 30-, 50-, 70-, 90-, 110-, 130-, 150- and 210-day-old boars, and performed histological and immunohistochemical analyses on testis sections. We first examined the development of spermatogenic cells and seminiferous tubules in porcine testes. Then, by immunofluorescence staining for marker proteins (AMH, SOX9, DBA, UCHL1, VASA, KIT, Ki67 and/or PCNA), we delved into the proliferative activity and development of Sertoli cells and of spermatogonial subtypes (pro-, undifferentiated and differentiating spermatogonia). Besides, by immunostaining for β-catenin and ZO-1, we studied the establishment of the blood-testis barrier in porcine testes.

**Conclusions:**

In this longitudinal study, we have systematically investigated the elaborate Sertoli cell and spermatogonial developmental patterns in pigs from the neonate to sexual maturity that have so far remained largely unknown. The findings not only extend the knowledge about spermatogenesis and testicular development in pigs, but also lay the theoretical groundwork for porcine breeding and rearing.

## Background

Spermatogenesis is an intricate developmental process during which the primitive male germ cells, i.e., spermatogonial stem cells (SSCs), undergo self-renewal and differentiation to generate eventually mature spermatozoa. This entire process is divided into three phases: the mitotic phase in which SSCs self-renew and differentiate, the meiotic phase in which spermatocytes undergo two rounds of division to form haploid spermatids, and the phase of spermiogenesis in which haploid round spermatids undergo elongation and transformation into spermatozoa [1]. Spermatogenesis occurs in seminiferous tubules within the testis, and the seminiferous tubules harbor Sertoli and germ cells, with the former being an integral component of the microenvironment that enables the continual production of spermatozoa thereby safeguarding lifelong male fertility.

Sertoli cells are the most important somatic cell type within the microenvironment that support and steer male germ cell development during spermatogenesis [2], and the number of Sertoli cells per testis is a key determinant of sperm generation. Typically, the Sertoli cell proliferation initiates prenatally and terminates before puberty, albeit highly variable among species [3, 4]. When they stop division and become mature, Sertoli cells are capable of supporting the entire spermatogenesis, which is along with the establishment of the blood-testis barrier [5]. The blood-testis barrier present between adjacent Sertoli cells divides the seminiferous epithelium into the basal and adluminal compartments, thereby creating an immune-privileged niche crucial to spermatogenic progress [3, 5, 6]. Depletion of Sertoli cells or disturbance of the blood-testis barrier within the seminiferous tubules has been reported to cause spermatogenic failure and consequently male infertility [5].

In neonatal testes, there are pro- and undifferentiated spermatogonia being the primary germ cell types at the onset of spermatogenesis. Prospermatogonia, also named gonocytes [7, 8], are derived from primordial germ cells (PGCs) prenatally. After birth, prospermatogonia progressively migrate from the center to the periphery of seminiferous cords, and upon localization at the basement membrane, a subset of prospermatogonia directly enters the differentiation trajectory, giving rise to the so-called first wave of spermatogenesis, whilst the others transform into undifferentiated spermatogonia consisting of SSCs and progenitors, which has clearly been reported in mice but remains to be demonstrated in higher mammals [7–10]. Only a rare fraction of undifferentiated spermatogonia is endowed with stem cell capacities thus perceived as SSCs [11–13], whereas the others, making up the majority of the undifferentiated population, are progenitors primed for differentiation, an irreversible process committed to meiosis and temporally orchestrated by the stages of the seminiferous epithelium in the testis [14, 15].

While the developmental progression of Sertoli cells and spermatogonia has been well established in mice, much less is known in other mammalian species including pigs. As a large domestic species, pigs share some similarity with humans in terms of anatomy, physiology and genetics, and are thus employed as model animals in mounting basic and translational research [16]. Moreover, like humans, pigs exhibit prolonged reproductive maturation [17, 18], being an alternative animal model for studies on reproductive biology. To acquire knowledge of Sertoli cell and spermatogonial development in pigs, here we collected as many as nine ages of Duroc porcine testes from the neonate to sexual maturity, and performed histological and immunohistochemical analyses on testis sections. We first examined the development of spermatogenic cells and seminiferous tubules in porcine testes. Then, by immunofluorescence staining for several marker proteins, we delved into the proliferative activity and development of Sertoli cells and of spermatogonial subtypes (pro-, undifferentiated and differentiating spermatogonia). Besides, we studied the establishment of the blood-testis barrier in porcine testes. Taken together, this longitudinal study tremendously extends the knowledge about spermatogenesis and testicular development in pigs, laying the theoretical groundwork for porcine breeding and rearing.

## Methods

### Testis sample collection

All testis samples were obtained from Duroc pigs raised in a farm affiliated with the Zhumei Porcine Breeding Corporation, Henan, China. Overall, we harvested nine ages of testes from twenty-seven Duroc pigs spanning from the neonate to sextual maturity, i.e., testes from 7-, 30-, 50-, 70-, 90-, 110-, 130-, 150- and 210-day-old boars, with testis samples at each age collected from three littermates. The surgical procedures were conducted by veterinarians and all subsequent animal experimentation was complied with and approved by the animal ethical committee of Northwest A&F University.

### Analyses of histology and the diameter of seminiferous cords/tubules

Upon castration, testes were maintained in Dulbecco’s phosphate buffered saline (DPBS) supplemented with 2% penicillin-streptomycin on ice and immediately transported to the laboratory. Testis tissue from the intermediate area between tunica albuginea and the transitional region was collected as previously described [19], and then cut into small fragments and fixed in Bouin’s solution or in 4% paraformaldehyde (PFA), followed by embedding in paraffin. Testis sections were then sliced at the thickness of 5 μm. After deparaffinization and rehydration, testis sections were subjected to hematoxylin-eosin (HE) staining, following the standard procedure. Later, testis sections were dehydrated and embedded in gum, and visualized under a Nikon i90 microscope (Nikon, Tokyo, Japan). The statistical analysis of the diameter of seminiferous cords/tubules at each age is based on 150 randomly selected round seminiferous cord/tubule cross-sections from three littermates, with 50 cord/tubule cross-sections per individual.

### Immunofluorescence staining and quantification of positive cells

Testis sections were deparaffinized, rehydrated, and alternatively subjected to heat-mediated antigen retrieval in sodium citrate buffer (0.01 mol/L, pH = 6.0). Then, testis sections were blocked with 10% donkey serum diluted in DPBS for 2 h at room temperature. After blocking, the sections were incubated with primary antibodies diluted in 10% donkey serum at 4 °C overnight. The primary antibodies utilized were as follows: DBA (1:100; RL-1032-2, Vector Laboratories, Burlingame, CA, United States), mouse anti-UCHL1 (1:300; ab8189, Abcam, Shanghai, China), rabbit anti-VASA (1:300; ab13840, Abcam, Shanghai, China), rabbit anti-KIT (1:200; 3074, Cell Signaling Technology, Shanghai, China), mouse anti-AMH (1:100; sc-166752, Santa Cruz Biotechnology, Shanghai, China), rabbit anti-SOX9 (1:600; ab185966, Abcam, Shanghai, China), rabbit anti-Ki67 (1:200; 27309–1-AP, Proteintech, Wuhan, Hubei, China), mouse anti-PCNA (1:200; 2586, Cell Signaling Technology, Shanghai, China), rabbit anti-β-catenin (1:200; 51067–2-AP, Proteintech, Wuhan, Hubei, China) and rabbit anti-ZO-1 (1:200; 21773–1-AP, Proteintech, Wuhan, Hubei, China). The isotype mouse or rabbit IgGs in place of the primary antibodies were used in negative controls. After washing on the next day, the testis sections were incubated with the corresponding secondary antibodies diluted in 10% donkey serum for 1 h at room temperature. The secondary antibodies utilized were donkey anti-rabbit AF488 (1:400; 34206ES60, Yeasen, Shanghai, China), donkey anti-rabbit AF594 (1:400; 34212ES60, Yeasen, Shanghai, China) and donkey anti-mouse AF488 (1:400; 34106ES60, Yeasen, Shanghai, China). After washing, the sections were counterstained with DAPI for 5 min at room temperature, and microscopy was then performed as previously described [20]. For quantification of positive cells per cross-section of seminiferous cords/tubules at each age, 150 randomly selected round seminiferous cord/tubule cross-sections from three littermates, with 50 cord/tubule cross-sections per individual, were analyzed.

### Statistics

To statistically analyze the significance of differences between two or multiple groups, the student’s *t*-test or one-way analysis of variance (ANOVA) followed by the least significant difference (LSD) method was employed, respectively. All quantitative data were demonstrated as the mean ± standard error of the mean (SEM), and differences were defined statistically significant at *P* < 0.05.

## Results

### Development of spermatogenic cells and seminiferous cords/tubules in porcine testes

**Fig. 1:**
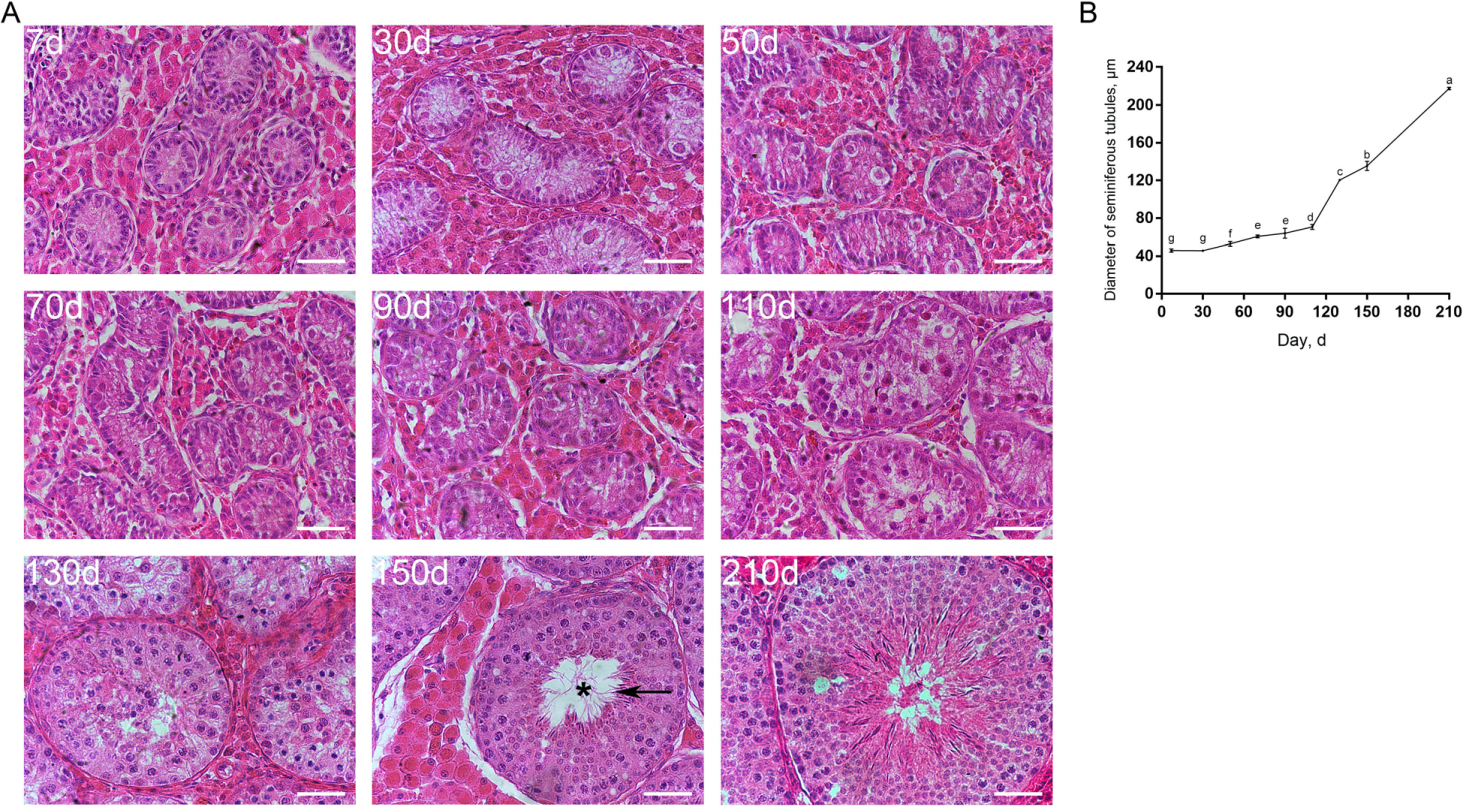
Development of spermatogenic cells and seminiferous cords/tubules in porcine testes. **A** HE staining of porcine testis sections at each age. The arrow points to spermatozoa, and the asterisk refers to seminiferous tubule lumen. Bar = 50 μm. **B** The diameter of seminiferous cords/tubules in porcine testes at each age. Data are presented as the mean ± SEM of three littermates, with 50 randomly selected round cord/tubule cross-sections analyzed per individual. Different letters indicate significant differences between groups (*P* < 0.05)

To systematically investigate the development of spermatogenic cells in pigs, we collected nine ages of Duroc porcine testes from the neonate to sexual maturity, i.e., testes from 7-, 30-, 50-, 70-, 90-, 110-, 130-, 150- and 210-day-old boars, and then performed histological analyses on testis sections. HE staining results showed that the seminiferous tubule lumen was discernable in testes since d 150 (Fig. 1A, asterisk). In addition, we could identify spermatocytes in testes since d 110 and round spermatids since d 130, but spermatozoa were not observed until d 150 (Fig. 1A, arrow). To acquire knowledge about the development of seminiferous cords/tubules in pigs, we analyzed the diameter of seminiferous cords/tubules at each age. The analysis revealed that the diameter of seminiferous cords/tubules went up since d 30, but the growing rate differed among ages. Specifically, the diameter of seminiferous cords/tubules showed a slow increase between d 30 and 110 but shot up between d 110 and 210 (Fig. 1B).

### Sertoli cell development in porcine testes

**Fig. 2:**
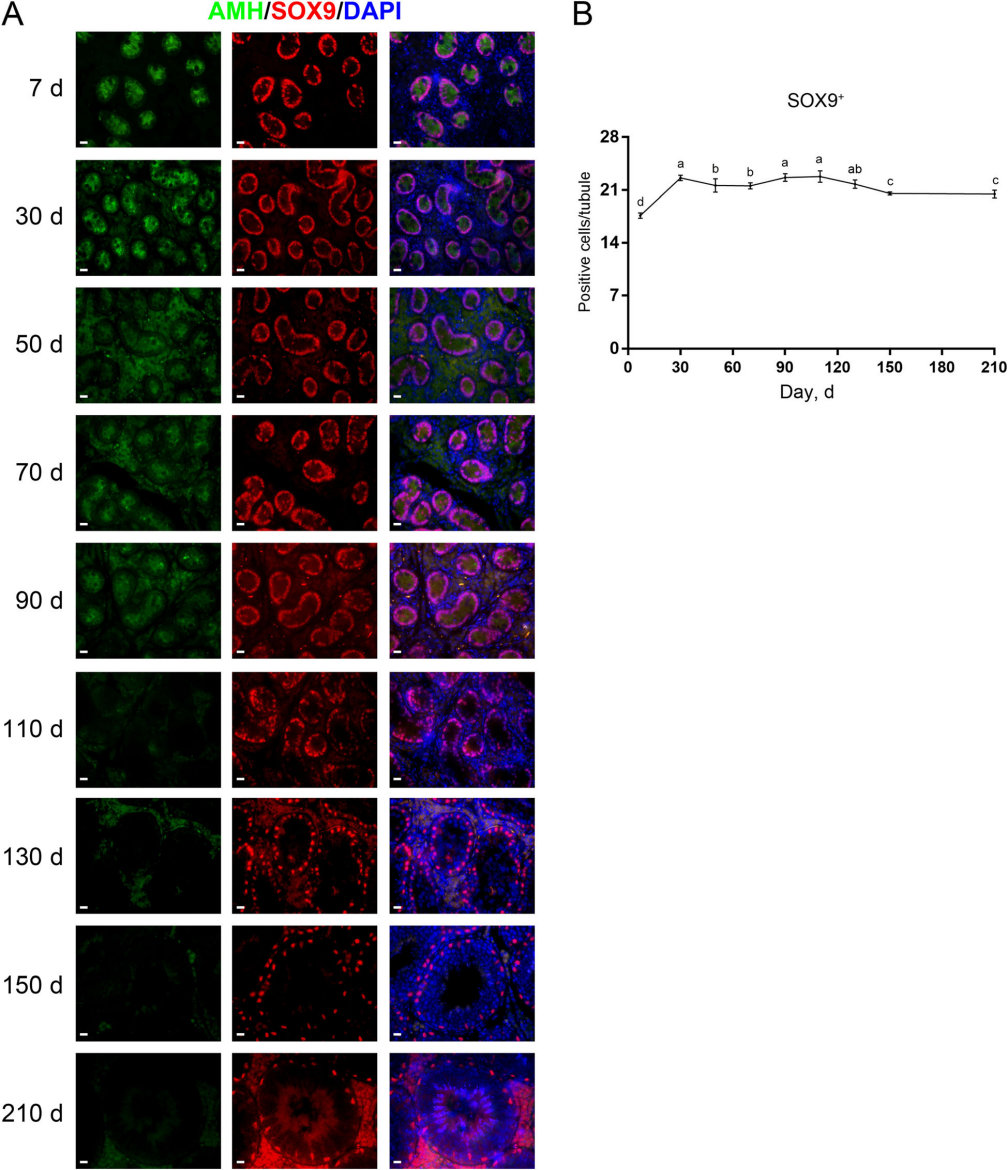
Immunostaining and quantification of AMH^+^ or SOX9^+^ cells in porcine testis sections. **A** AMH and SOX9 immunostaining of porcine testis sections at each age. Bar = 10 μm. **B** The numbers of SOX9^+^ cells per cross-section of seminiferous cords/tubules in porcine testes at each age. Data are presented as the mean ± SEM of three littermates, with 50 round cord/tubule cross-sections analyzed per individual. Different letters indicate significant differences between groups (*P* < 0.05)

Next, we explored the development of Sertoli cells in pigs. To identify Sertoli cells we applied antibodies against anti-Müllerian hormone (AMH) or SOX9. AMH is a member of the TGF-β family specifically expressed in immature Sertoli cells [21], whereas SOX9 serves as a whole-stage marker for Sertoli cells [22]. By double immunofluorescence staining for AMH and SOX9 on testis sections, we identified that the AMH staining was present in porcine testes from d 7 to 90 but absent since d 110, suggesting that porcine Sertoli cells become mature at 110 days of age. Unlike AMH, the staining for SOX9 persisted in porcine testes at all ages (Fig. 2A). Since it is tricky to quantify AMH^+^ cells due to the diffused staining of AMH, we then quantified the SOX9^+^ cells per seminiferous cord/tubule cross-section. We found that the numbers of SOX9^+^ cells substantially increased between d 7 and 30, and then marginally fluctuated (Fig. 2B).

### The proliferative activity of Sertoli cells in porcine testes

**Fig. 3:**
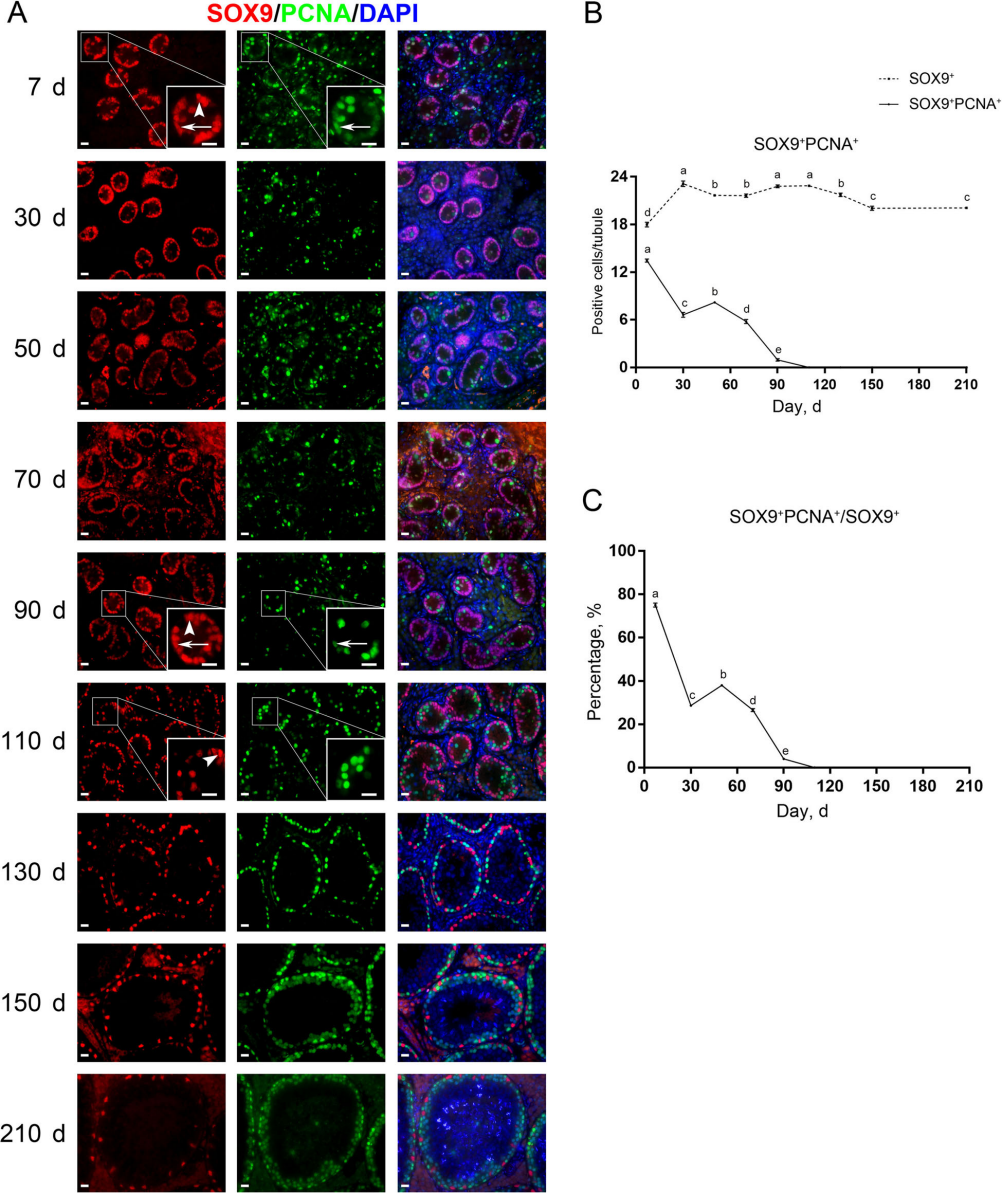
Immunostaining and quantification of SOX9^+^ and PCNA^+^ cells in porcine testis sections. **A** SOX9 and PCNA immunostaining of porcine testis sections at each age. Arrows and arrowheads point to SOX9^+^PCNA^+^ and SOX9^+^PCNA^−^ cells, respectively. Bar = 10 μm. **B** The numbers of SOX9^+^ and SOX9^+^PCNA^+^ cells per cross-section of seminiferous cords/tubules in porcine testes at each age. **C** The percentages of SOX9^+^ cells positive for PCNA in porcine testes at each age. Data are presented as the mean ± SEM of three littermates, with 50 round cord/tubule cross-sections analyzed per individual. Different letters indicate significant differences between groups (*P* < 0.05)

To learn about the proliferative activity of Sertoli cells in pigs, we then performed co-staining for SOX9 and PCNA on testis sections. Co-immunofluorescence staining results showed that both SOX9^+^PCNA^+^ and SOX9^+^PCNA^−^ cells were present in porcine testes from d 7 to 90 (Fig. 3A, arrows), but that only SOX9^+^PCNA^−^ cells could be observed since d 110 (Fig. 3A, arrowheads), suggesting that a subpopulation of immature Sertoli cells is in the active cell cycle and that mature Sertoli cells, as indicated by loss of AMH staining, are not. Quantification of cells positive for SOX9 and PCNA per cross-section of seminiferous cords/tubules revealed that both the numbers of SOX9^+^PCNA^+^ cells and the ratios of proliferating SOX9^+^ cells showed generally downward trends with age, with no SOX9^+^PCNA^+^ cells since d 110 (Fig. 3B and C).

### Establishment of the blood-testis barrier in porcine testes

**Fig. 4:**
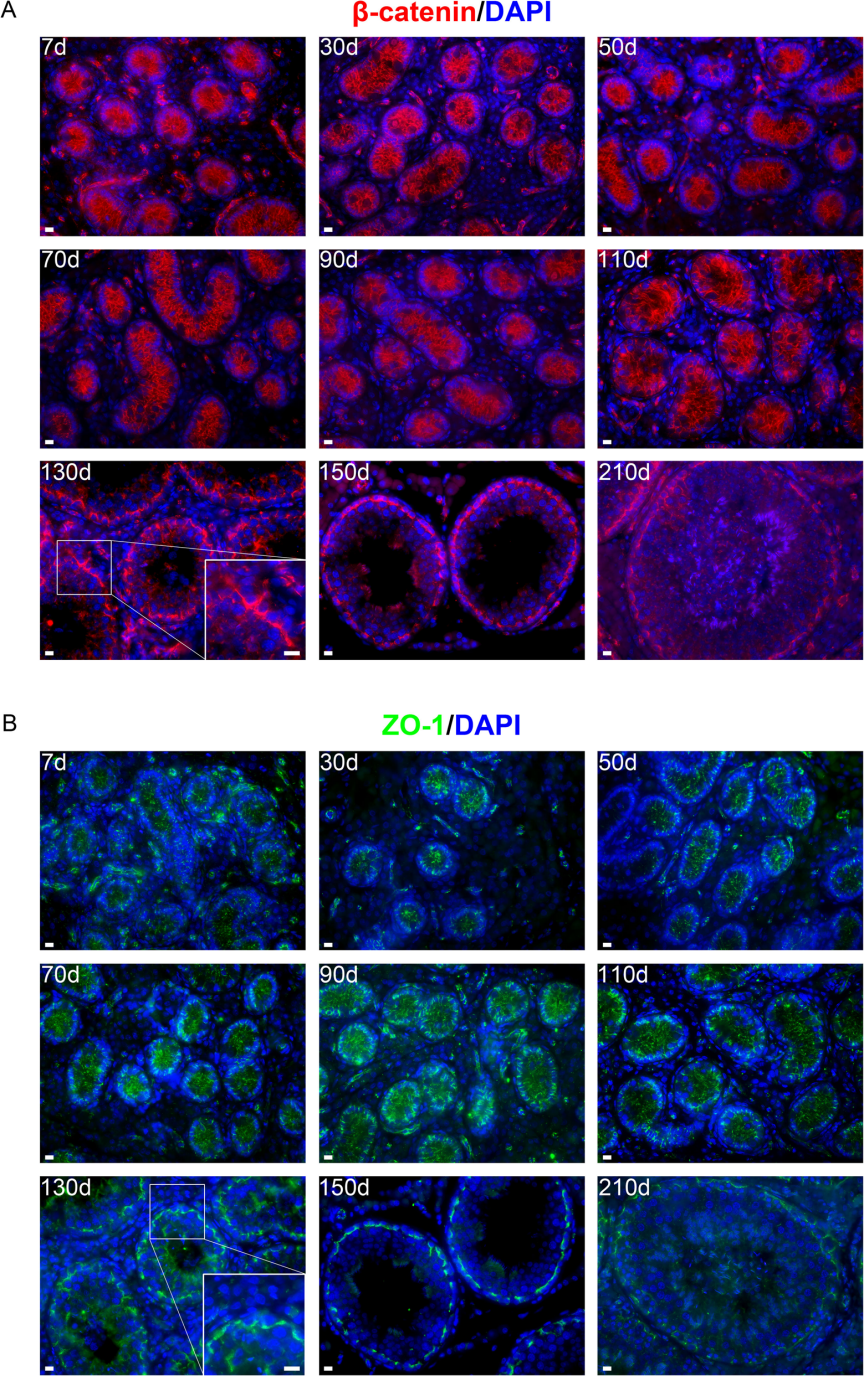
Immunostaining for β-catenin (**A**) and ZO-1 (**B**) on porcine testis sections. Bar = 10 μm

The blood-testis barrier, constructed by specialized junctional protein complexes between adjacent Sertoli cells, creates an immune-privileged niche crucial to spermatogenic progress [3, 5, 6]. Despite the importance, little is known about the blood-testis barrier establishment in pigs. To this end, we performed immunofluorescence staining for two blood-testis barrier structural components, i.e., β-catenin and ZO-1, on testis sections. β-catenin is a component of the testis-specific adherens junction, while ZO-1 is a major component of the tight junction that divides the seminiferous epithelium into basal and adluminal compartments [23, 24]. We identified that both β-catenin and ZO-1 showed discrete staining patterns from d 7 to 110, but that these two proteins displayed conjunctive staining patterns from d 130 onwards (Fig. 4A and B), suggesting that the blood-testis barrier forms at 130 days of age in pigs.

### Development of pro- and undifferentiated spermatogonia in porcine testes

**Fig. 5:**
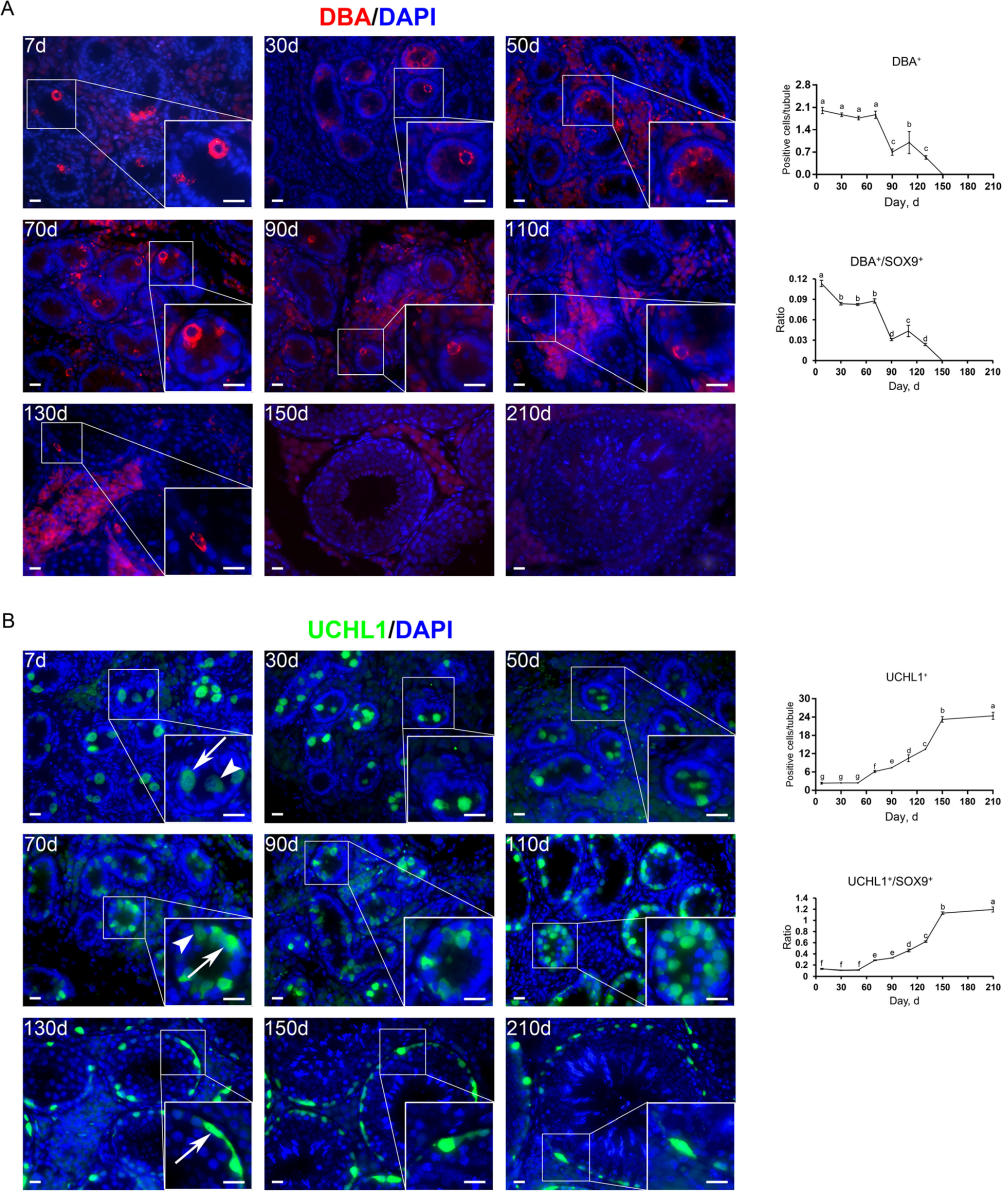
Immunostaining and quantification of DBA^+^ or UCHL1^+^ cells in porcine testis sections. **A** Left: DBA immunostaining of porcine testis sections at each age. Bar = 10 μm. Right: the numbers of DBA^+^ cells (upper) and the ratios of DBA^+^ to SOX9^+^ cells (lower) per cross-section of seminiferous cords/tubules in porcine testes at each age. **B** Left: UCHL1 immunostaining of porcine testis sections at each age. Arrows and arrowheads point to UCHL1^high^ and UCHL1^low^ cells, respectively. Bar = 10 μm. Right: the numbers of UCHL1^+^ cells (upper) and the ratios of UCHL1^+^ to SOX9^+^ cells (lower) per cross-section of seminiferous cords/tubules in porcine testes at each age. Data are presented as the mean ± SEM of three littermates, with 50 round cord/tubule cross-sections analyzed per individual. Different letters indicate significant differences between groups (*P* < 0.05)

Subsequently, to probe the development of spermatogonia in pigs, we employed marker proteins to distinguish spermatogonial subtypes. To identify pro- and undifferentiated spermatogonia we applied *Dolichos biflorus* agglutinin (DBA) and UCHL1. DBA is a lectin that shows specific attachment to primitive male germ cells, i.e., prospermatogonia and early undifferentiated spermatogonia, in neonatal porcine testes [25]. Immunofluorescence staining results showed that at young ages most DBA^+^ cells were present at the center of seminiferous cords, and that with age, increasing DBA^+^ cells migrated to the basement membrane, with all DBA^+^ cells located at the basement membrane since d 130 (Fig. 5A, left). Quantification of DBA^+^ cells per cross-section of seminiferous cords/tubules revealed that the highest numbers of DBA^+^ cells were present between d 7 and 70, and the highest ratio of DBA^+^ to SOX9^+^ cells was at d 7. Later, DBA^+^ cells gradually vanished, with no DBA^+^ cells observed since d 150 (Fig. 5A, right).

UCHL1, also known as PGP9.5, is exclusively expressed in porcine pro- and undifferentiated spermatogonia [26, 27]. Immunofluorescence staining for UCHL1 showed that UCHL1^+^ cells were present in porcine testes at all ages. Technically, at young ages UCHL1^+^ cells could be identified at the center or periphery of seminiferous cords, but all UCHL1^+^ cells, same as the entire DBA^+^ population, were located at the basement membrane since d 130. At all ages, some cells stained heavily for UCHL1 (Fig. 5B, arrows), while others weakly stained (Fig. 5B, arrowheads). Quantification of UCHL1^+^ cells per cross-section of seminiferous cords/tubules revealed that both the numbers of UCHL1^+^ cells and the ratios of UCHL1^+^ to SOX9^+^ cells consistently increased since d 50 and culminated at 210 days of age (Fig. 5B, right).

### Development of differentiating spermatogonia in porcine testes

**Fig. 6:**
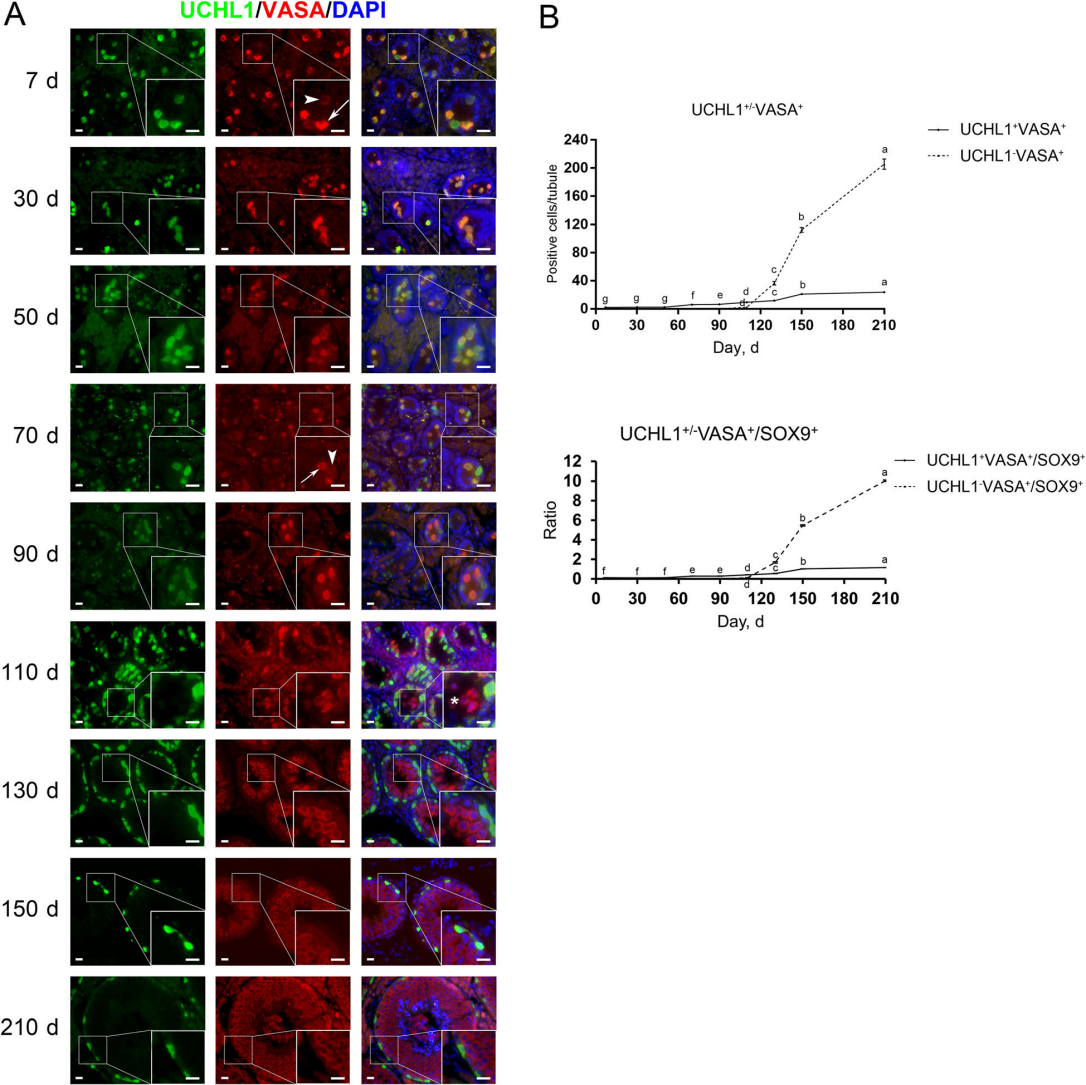
Immunostaining and quantification of UCHL1^+/−^VASA^+^ cells in porcine testis sections. **A** UCHL1 and VASA immunostaining of porcine testis sections at each age. Arrows and arrowheads point to VASA^high^ and VASA^low^ cells, respectively, and the asterisk refers to UCHL1^−^VASA^+^ cells. Bar = 10 μm. **B** The numbers of UCHL1^+/−^VASA^+^ cells (upper) and the ratios of UCHL1^+/−^VASA^+^ to SOX9^+^ cells (lower) per cross-section of seminiferous cords/tubules in porcine testes at each age. Data are presented as the mean ± SEM of three littermates, with 50 round cord/tubule cross-sections analyzed per individual. Different letters indicate significant differences between groups (*P* < 0.05)

To pinpoint the specific expression of UCHL1 in pro- and undifferentiated spermatogonia and the timepoint of spermatogonial differentiation in pigs, we next performed double immunostaining for UCHL1 and VASA or KIT on testis sections. VASA, also known as DDX4 or MVH, is a conserved pan-germ cell marker in testes [28, 29]. Immunofluorescence staining results showed that UCHL1 shared an identical localization pattern with VASA in testes from d 7 to 90, i.e., UCHL1^+^ cells were also positive for VASA, and vice versa. UCHL1^−^VASA^+^ cells could be observed since d 110 (Fig. 6A, asterisk), suggesting the emergence of differentiating spermatogonia. Similar to that of UCHL1, some cells stained heavily for VASA (Fig. 6A, arrows), while others weakly stained (Fig. 6A, arrowheads). Typically, cells strongly expressing UCHL1 stained weakly for VASA, whereas those with weak or no expression of UCHL1 heavily stained for VASA, in accordance with a previous report [27] and suggesting the upregulation of VASA during spermatogonial differentiation. Quantification of cells positive for UCHL1 and VASA per cross-section of seminiferous cords/tubules revealed that both UCHL1^−^VASA^+^ cells and the ratios of UCHL1^−^VASA^+^ to SOX9^+^ cells showed a sharp rise since d 110 (Fig. 6B).

**Fig. 7:**
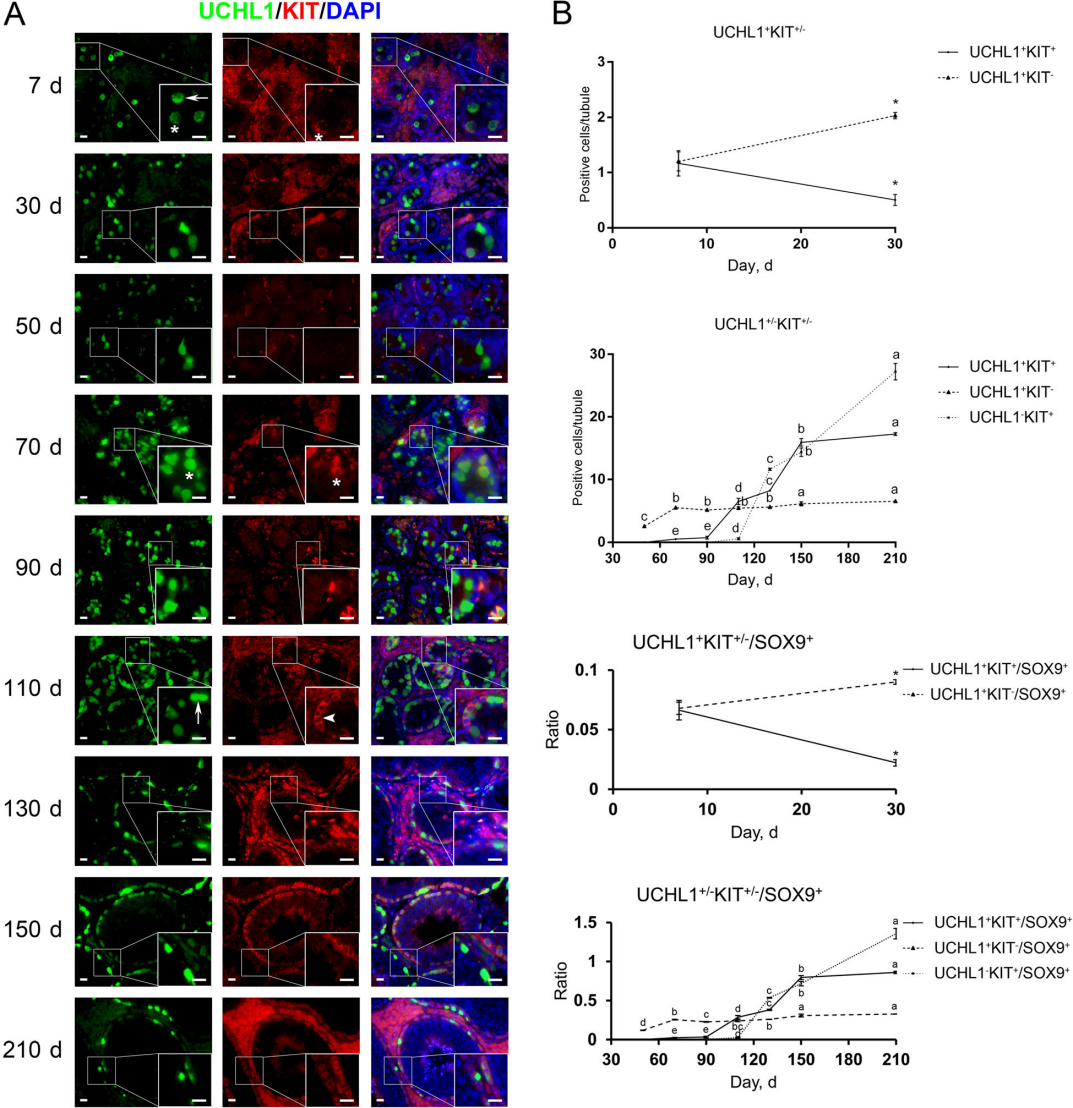
Immunostaining and quantification of UCHL1^+/−^KIT^+/−^ cells in porcine testis sections. **A** UCHL1 and KIT immunostaining of porcine testis sections at each age. Arrows, arrowheads and asterisks point to UCHL1^+^KIT^−^, UCHL1^−^KIT^+^ and UCHL1^+^KIT^+^ cells, respectively. Bar = 10 μm. **B** The upper panels: the numbers of UCHL1^+/−^KIT^+/−^ cells per cross-section of seminiferous cords/tubules in porcine testes at each age. The lower panels: the ratios of UCHL1^+/−^KIT^+/−^ to SOX9^+^ cells per cross-section of seminiferous cords/tubules in porcine testes at each age. Data are presented as the mean ± SEM of three littermates, with 50 randomly selected round cord/tubule cross-sections analyzed per individual. Asterisks and different letters indicate significant differences between groups (*P* < 0.05)

KIT is widely known as a conserved marker protein specifically expressed in differentiating spermatogonia [30–35]. Surprisingly, by co-staining for UCHL1 and KIT we identified KIT^+^ cells in porcine testes at all ages except d 50, and that KIT^+^ cells were also positive for UCHL1 from d 7 to 90. UCHL1^−^KIT^+^ cells were discernable since d 110 (Fig. 7A, arrowhead), while UCHL1^+^KIT^−^ cells could be observed in porcine testes at all ages (Fig. 7A, arrows). Most KIT^+^ cells showed large size and round morphology and were located at the center of seminiferous cords at 7 and 30 days of age, suggesting their prospermatogonial property. Quantification of cells positive for UCHL1 and KIT revealed that 49.96% ± 6.05% of UCHL1^+^ cells were positive for KIT at 7 days of age, with only 26.74% ± 3.40% of UCHL1^+^ cells positive for KIT at d 30. Despite no KIT^+^ cells at 50 days of age, UCHL1^+^KIT^+^ cells reemerged at d 70 and their numbers shot up since d 90 (Fig. 7A, asterisks). UCHL1^−^KIT^+^ cells, which are supposed to represent differentiating spermatogonia, emerged at d 110 and then their numbers rapidly increased (Fig. 7B). Together, our results demonstrate that KIT is expressed not only in differentiating spermatogonia but also in a subpopulation of primitive male germ cells in particular prospermatogonia in pigs.

### The proliferative activity of spermatogonia in porcine testes

**Fig. 8:**
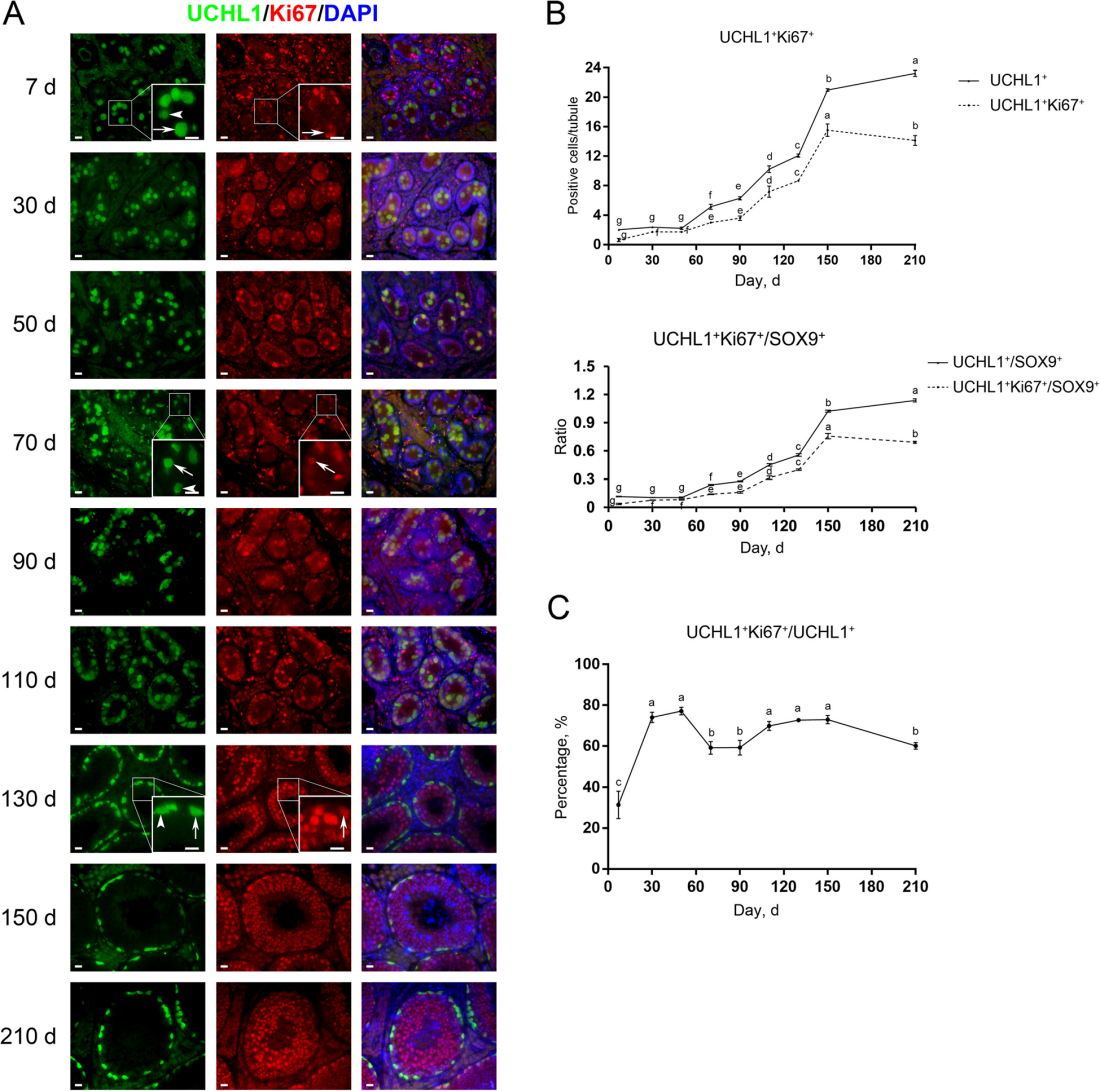
Immunostaining and quantification of UCHL1^+^Ki67^+^ cells in porcine testis sections. **A** UCHL1 and Ki67 immunostaining of porcine testis sections at each age. Arrows and arrowheads point to UCHL1^+^Ki67^+^ and UCHL1^+^Ki67^−^ cells, respectively. Bar = 10 μm. **B** The numbers of UCHL1^+^Ki67^+^ cells (upper) and the ratios of UCHL1^+^Ki67^+^ to SOX9^+^ cells (lower) per cross-section of seminiferous cords/tubules in porcine testes at each age. **C** The percentages of UCHL1^+^ cells positive for Ki67 in porcine testes at each age. Data are presented as the mean ± SEM of three littermates, with 50 round cord/tubule cross-sections analyzed per individual. Different letters indicate significant differences between groups (*P* < 0.05)

To gain knowledge about the proliferative activity of spermatogonial subpopulations in pigs, we then performed co-staining for UCHL1 and Ki67 or for KIT and PCNA on testis sections. Ki67 and PCNA are markers of cells in the active cell cycle and are expressed in nuclei of proliferating cells [34, 36]. Co-immunofluorescence staining results showed that UCHL1^+^Ki67^+^ cells were present at the center and/or periphery of seminiferous cords/tubules in porcine testes at all ages (Fig. 8A, arrows), and that both UCHL1^+^Ki67^+^ and UCHL1^+^Ki67^−^ cells were present at each age (Fig. 8A, arrowheads). Quantification of cells positive for UCHL1 and Ki67 per cross-section of seminiferous cords/tubules revealed that both the numbers of UCHL1^+^Ki67^+^ cells and the ratios of UCHL1^+^Ki67^+^ to SOX9^+^ cells consistently grew with age, peaked at d 150, and marginally declined at d 210 (Fig. 8B). Notably, while the majority of prospermatogonia were quiescent at d 7, as indicated by only around 30% of UCHL1^+^ cells being positive for Ki67, at all other ages predominant UCHL1^+^Ki67^+^ cell populations could be observed (Fig. 8C), suggesting the proliferative property of early developing spermatogonia.

**Fig. 9:**
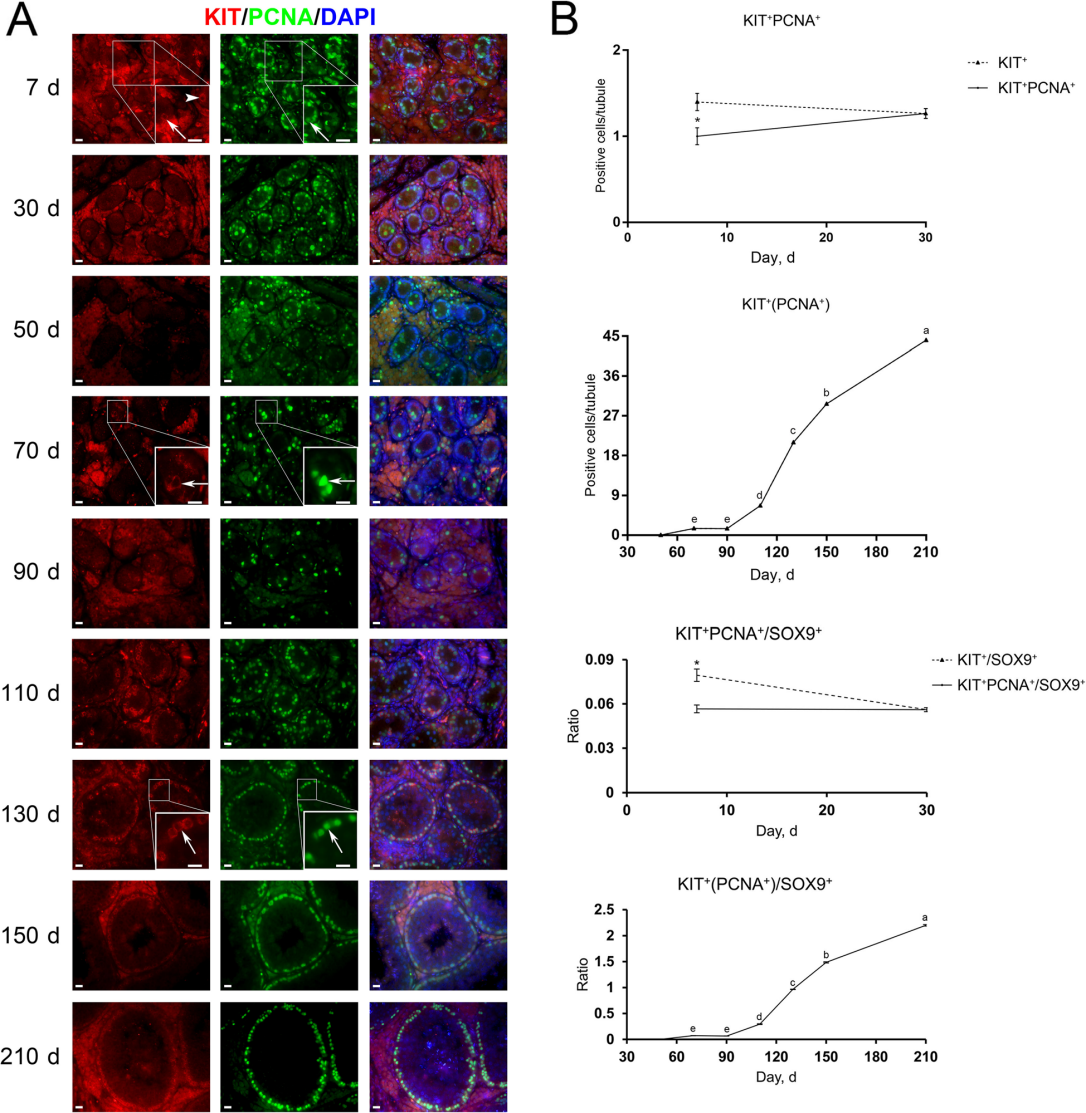
Immunostaining and quantification of KIT^+^PCNA^+^ cells in porcine testis sections. **A** KIT and PCNA immunostaining of porcine testis sections at each age. Arrows and arrowheads point to KIT^+^PCNA^+^ and KIT^+^PCNA^−^ cells, respectively. Bar = 10 μm. **B** The upper panels: the numbers of KIT^+^PCNA^+^ cells per cross-section of seminiferous cords/tubules in porcine testes at each age. The lower panels: the ratios of KIT^+^PCNA^+^ to SOX9^+^ cells per cross-section of seminiferous cords/tubules in porcine testes at each age. Data are presented as the mean ± SEM of three littermates, with 50 round cord/tubule cross-sections analyzed per individual. Different letters indicate significant differences between groups (*P* < 0.05)

Different from the co-staining result of UCHL1 and Ki67, at d 7 the majority (74.33% ± 11.16%) of KIT^+^ cells were positive for PCNA (Fig. 9A, arrows), and KIT^+^PCNA^−^ cells could be observed only at this age (Fig. 9A, arrowhead). In other words, from d 30 onwards, KIT^+^ cells were all positive for PCNA, suggesting that at least all differentiating spermatogonia in pigs are actively proliferating, consistent with that in mice [34, 37]. KIT^+^PCNA^+^ cells were also observed at both the center and periphery of seminiferous cords/tubules in porcine testes. Quantification of cells positive for KIT and PCNA per cross-section of seminiferous cords/tubules revealed that despite no KIT^+^ cells at 50 days of age, KIT^+^PCNA^+^ cells reemerged at d 70, and that both their numbers and the ratios of KIT^+^PCNA^+^ to SOX9^+^ cells jumped since d 90 and culminated at d 210 (Fig. 9B).

## Discussion

In the present study, we collected and performed morphological and immunohistochemical analyses on nine ages of Duroc porcine testes from the neonate to sexual maturity, with an aim to gain further insights into the development of testicular cells in particular Sertoli cells and spermatogonia in pigs. Previous studies have demonstrated that the testis size grows with age, and that the diameter of seminiferous tubules generally remains stable between the neonate and the 2 months of age in pigs [38–40]. In addition, it has been reported that in pigs each spermatogenic cycle lasts for approximately 8.6 d and the full spermatogenic process lasts for around 40 d [41], and that somatic cell proliferation and seminiferous tubule maturation in postnatal porcine testes are not random [19]. Our results showed that the diameter of seminiferous tubules went up since d 30, with a slow increase between d 30 and 110 and acceleration from d 110 onwards. The underlying reason might be that boars are prepared to enter puberty after 110 days of age. Typically, boars arrive at puberty between 5 and 6 months and reach sexual maturity between 6 and 7 months, while full maturity can be as late as 7–8 months [17, 19, 41–43], which, however, may vary among strains and individuals, and the feeding condition is also a contributor to this variability. Once arriving at puberty, advanced spermatogenic cells, such as various stages of spermatocytes, round/elongating spermatids and spermatozoa, and seminiferous tubule lumen are discernable. Here, while spermatocytes and round spermatids were identified in porcine testes since d 110 and d 130 respectively, spermatozoa and seminiferous tubule lumen were not observed until d 150, suggesting that pigs can reach puberty at 150 days of age.

In mice, immature Sertoli cells proliferate for a short period of time after birth, and typically reach the terminal differentiation status at approximately post-natal d 15 [3]. Despite this, little is known regarding when porcine Sertoli cells become mature. To this end, we investigated the expression of AMH and SOX9 which mark the immature and whole-stage Sertoli cells, respectively [21, 22]. We found that AMH staining vanished in porcine testes since d 110, suggesting that porcine Sertoli cells can become mature at 110 days of age. Meanwhile, this was accompanied by loss of proliferating Sertoli cells, suggesting that mature Sertoli cells are not in the active cell cycle. Interestingly, recent studies have shown that adult Sertoli cells in a specific region between the seminiferous tubule and rete testis, called the transition region, retain immature properties and consequently the capability of proliferation [44]. Future studies are needed to acquire more knowledge in this respect.

The blood-testis barrier plays multiple important roles such as protection of spermatozoa from autoimmunity, which is based on its structural integrity. Upon the destruction of the blood-testis barrier, spermatogenesis is also disturbed and even comes to a standstill [45]. The principal component of the blood-testis barrier is tight junctions. In mice, an early report showed that tight junctions between Sertoli cells developed between post-natal d 10 and 16 [46], and subsequent studies demonstrated that the blood-testis barrier formed at around post-natal d 15 [47]. Nevertheless, little is known about the blood-testis barrier establishment in pigs. To this end, we investigated the expression of β-catenin and ZO-1 which are the major components of adherens junctions and tight junctions, respectively [23, 24]. We identified that both β-catenin and ZO-1 displayed conjunctive staining patterns from d 130 onwards, suggesting that the blood-testis barrier can form at 130 days of age in pigs, later than Sertoli cell maturation (d 110). Meanwhile, establishment of the blood-testis barrier in porcine testes at d 130 was accompanied by emergence of various stages of spermatocytes and round spermatids, corroborating that the blood-testis barrier, dividing the seminiferous epithelium into the basal and adluminal compartments in which early (spermatogonia and preleptotene spermatocytes) and late spermatogenic cells (advanced spermatocytes and spermatids) are located respectively [4], is essential for the normal meiotic progression and continuous sperm production. In future, the functionality or integrity evaluation, such as transmission electron microscopy or intercellular tracers, would provide more information about the time when the blood-testis barrier is actually functional.

Prospermatogonia can be categorized into three subgroups: mitotic (M), transitional 1 and 2 prospermatogonia (T1 and T2). Before birth, PGCs, once destined to males, proliferate for a couple of days in mice or weeks in humans and differentiate into prospermatogonia (M-prospermatogonia). Then, prospermatogonia enter the G_0_/G_1_ phase of cell cycle and stay quiescent (T1-prospermatogonia). After birth, prospermatogonia resume proliferation and migrate to the basement membrane (T2-prospermatogonia) [7, 10, 48, 49]. In mice, a subpopulation of prospermatogonia subsequently transits into undifferentiated spermatogonia comprising SSCs and progenitors, whereas the others directly develop into differentiating spermatogonia responsible for the first wave of spermatogenesis [7–10]. Prospermatogonial migration to the basement membrane and transition to undifferentiated/differentiating spermatogonia is, however, not instant, which initiates at about post-natal d 3 and finalizes between d 5 and 7 in mice [8, 50, 51]. In pigs, previous studies have reported that transformation of prospermatogonia to spermatogonia initiates at approximately 2 months after birth [25, 40, 52], but it remains elusive when this transition finalizes. Here, we identified that increasing DBA^+^ and UCHL1^+^ cells, characterizing pro- and/or undifferentiated spermatogonia, migrated to the basement membrane with age, with all DBA^+^ and UCHL1^+^ cells located at the basement membrane since d 130, suggesting that prospermatogonial migration and transition may finalize at 130 days of age in pigs. Indeed, it seems somewhat late to have porcine prospermatogonial migration and transition finalized at d 130, an age close to puberty. Yet, the majority of prospermatogonia should have finalized the migration and transition earlier, as only few prospermatogonia were present at the center of seminiferous cords at ages close to d 130. These luminal prospermatogonial population might include the aberrantly developed germ cells that would be cleared from the testis.

To study the onset of spermatogonial differentiation in pigs, we performed co-staining for UCHL1 and VASA on porcine testis sections. UCHL1^−^VASA^+^ cells were observed in porcine testes from d 110 onwards. Since UCHL1 exclusively marks porcine pro- and undifferentiated spermatogonia [27], emergence of UCHL1^−^VASA^+^ cells could indicate the onset of spermatogonial differentiation. For validation, we further performed co-staining for UCHL1 and KIT, as KIT is a conserved marker protein expressed in differentiating spermatogonia [30–35]. Intriguingly, we identified that KIT was expressed not only in porcine differentiating spermatogonia (UCHL1^−^KIT^+^) but also in a subpopulation of primitive male germ cells in particular prospermatogonia (UCHL1^+^KIT^+^). In post-natal mouse testes, KIT is expressed in differentiating spermatogonia. However, unlike that in mice, in post-natal rat testes the KIT protein expression could be detected in prospermatogonia as well as in differentiating spermatogonia [53–55], suggesting that KIT is required for rat (pro-)spermatogonial migration and differentiation. Thus, our study, showing the distinctive expression pattern of KIT in porcine testes, together with the previous findings in rodents, suggest the divergent features during male germline development among species, and that cautions are needed when taking advantage of this surface marker to enrich porcine differentiating spermatogonia by means such as magnetic-activated cell sorting (MACS) and fluorescence-activated cell sorting (FACS). In addition, we observed that most UCHL1^+^KIT^−^ cells stained heavily for UCHL1, whereas most UCHL1^+^KIT^+^ cells weakly stained for UCHL1, consistent with a previous report that the asymmetric distribution of UCHL1 in porcine undifferentiated spermatogonia is related to spermatogonial fate determination [27]. Unlike the burgeoning UCHL1^−^KIT^+^ cells since d 110, which are indicative of differentiating spermatogonia, the numbers of UCHL1^+^KIT^−^ cells per cross-section of seminiferous cords/tubules in porcine testes generally remained stable since d 70 (5.8 ± 0.5), suggesting that these cells are the reservoir of spermatogonia likely to include SSCs.

Spermatogenesis is a continuous process dependent on spermatogonial proliferation and differentiation. We then investigated the proliferative activity of spermatogonial subpopulations by performing co-staining for UCHL1 and Ki67 or for KIT and PCNA on porcine testis sections. As two prevalent cell proliferation markers, Ki67 is expressed in all phases of the cell cycle except G_0_ [56], while PCNA is synthesized in early G_1_ and S phases and its expression gradually increases in G_1_, peaks in S and decreases in G_2_/M [57]. We identified that most pro−/undifferentiated spermatogonia were quiescent at d 7 and resumed proliferation between d 30 and 50, which, however, was not accompanied by the significantly increased cell number at this age, probably due to the apoptosis of a fraction of pro/undifferentiated spermatogonia [58, 59]. From d 70 onwards, the numbers of pro/undifferentiated spermatogonia consistently grew, along with high ratios of these cells in the active cell cycle. Notably, both luminal and peripheral UCHL1^+^Ki67^+^ cells could be observed, corroborating that prospermatogonial proliferation and migration are independent events during male germline development [10, 49, 50]. Besides, KIT^+^ cells were all in the active cell cycle at all ages except d 7, same as the mouse differentiating spermatogonia that are all actively proliferating but different from their human counterparts that just over half do so [34, 37].

**Fig. 10:**
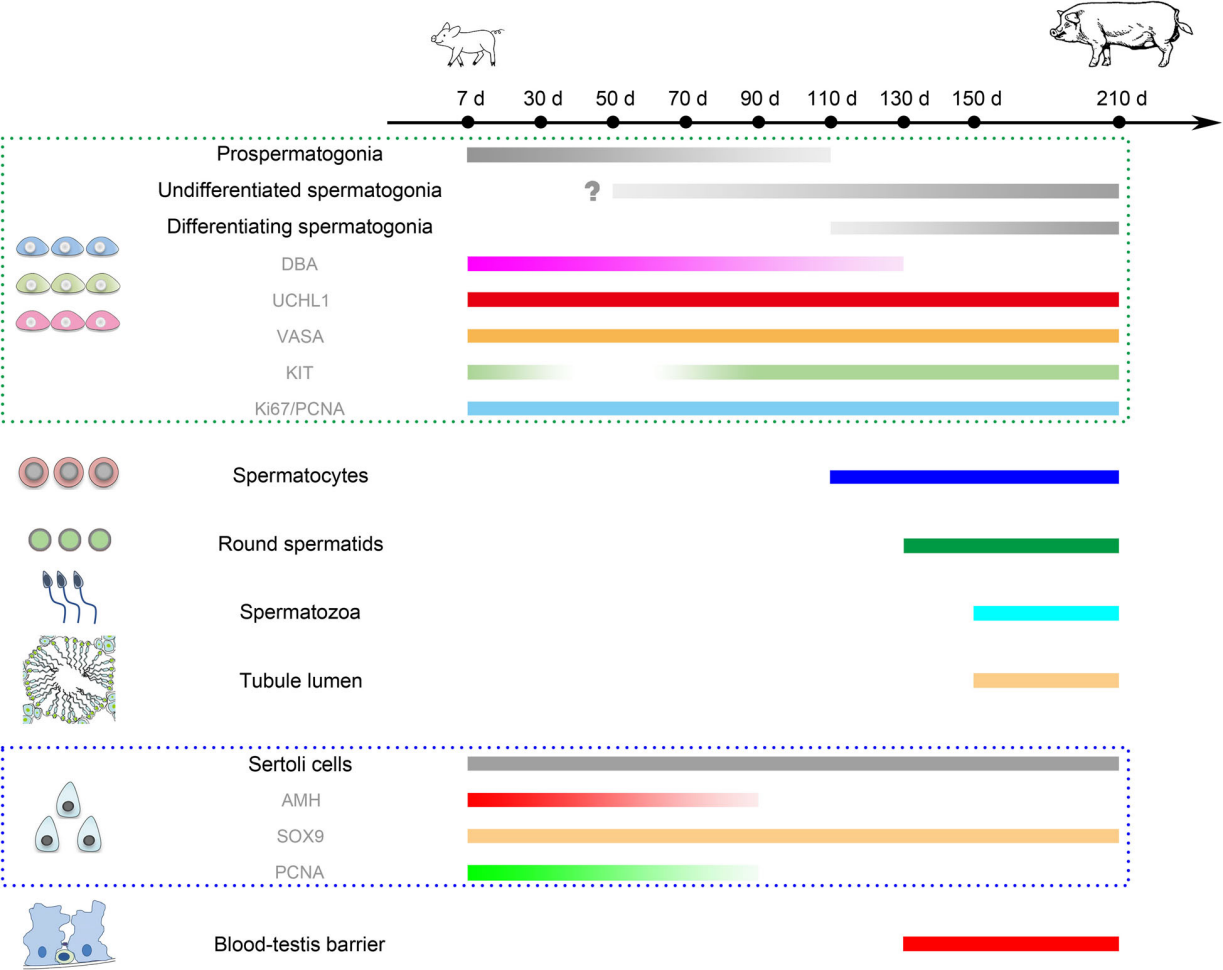
A schematic overview illustrating the primary findings in this study. The question mark (?) refers to the content that has been reported previously but not addressed in the current study

To sum up, in the present study, by performing morphological and immunohistochemical analyses on as many as nine ages of Duroc porcine testes from the neonate to sexual maturity, we systematically investigated testicular development, in particular Sertoli cell and spermatogonial development in pigs (Fig. 10). Indeed, it has to be acknowledged that porcine testicular development can vary among strains and individuals, with the feeding condition and climate being additional contributors to the variability. Nevertheless, this longitudinal study greatly adds to the body of knowledge about porcine spermatogenesis and testicular development, and lays the theoretical foundation for porcine breeding as well as application of assisted reproductive technology to pigs, with a long-term goal to enhance the fecundity and productivity of this important domestic species.

## Conclusions

In this longitudinal study, we have systematically investigated the elaborate Sertoli cell and spermatogonial developmental patterns in pigs from the neonate to sexual maturity that have so far remained largely unknown. The findings not only extend the knowledge about spermatogenesis and testicular development in pigs, but also lay the theoretical groundwork for porcine breeding and rearing.

## Data Availability

All data supporting our findings are included in the manuscript.

## Abbreviations

AMH: Anti-Müllerian hormone
ANOVA: Analysis of variance
DBA: *Dolichos biflorus* agglutinin
DPBS: Dulbecco’s phosphate buffered saline
FACS: Fluorescence-activated cell sorting
HE: Hematoxylin-eosin
LSD: Least significant difference
M: Mitotic
MACS: Magnetic-activated cell sorting
PFA: Paraformaldehyde
PGCs: Primordial germ cells
SEM: The mean ± standard error of the mean
SSCs: Spermatogonial stem cells
T1: Transitional 1
T2: Transitional 2
